# Cardioprotective effect of *Saussurea involucrata* injection against Doxorubicin-induced cardiotoxicity by network pharmacology analysis and experimental verification: *Saussurea involucrata* injection attenuates DIC

**DOI:** 10.3724/abbs.2024170

**Published:** 2024-12-04

**Authors:** Ding Wang, Yu Jin, Mengyu Yang, Yajing Xue, Xiaotong Zhang, Yanli Guo, Xinzhi Li, Ketao Ma

**Affiliations:** 1 Key Laboratory of Xinjiang Endemic and Ethnic Diseases Ministry of Education Shihezi University School of Medicine Shihezi 832003 China; 2NHC Key Laboratory of Prevention and Treatment of Central Asia High Incidence Diseases First Affiliated Hospital Shihezi University School of Medicine Shihezi 832003 China; 3 Department of Pathophysiology Shihezi University School of Medicine Shihezi 832003 China; 4Department of Physiology Shihezi University School of Medicine Shihezi 832003 China

**Keywords:** Doxorubicin, cardiotoxicity, *Saussurea involucrata* injection, network pharmacology, P53

## Abstract

Doxorubicin (Dox) is widely utilized in the clinical treatment of various cancers. Despite its efficacy, Dox induces numerous adverse effects in humans with significant cardiotoxicity, posing a major limitation to its use. *Saussurea involucrata* injection (SII), derived from *Saussurea involucrata*, exhibits notable anti-inflammatory and anti-oxidative stress properties. However, its potential protective effects against Dox-induced cardiotoxicity (DIC) remain unexplored. In this study, we investigate the ability of SII to mitigate DIC and elucidate the underlying mechanisms through experimental research and network pharmacology analysis. Results from both *in vitro* and *in vivo* experiments reveal that SII treatment significantly improves Dox-induced cardiac dysfunction, reducing pathological alterations and fibrosis in cardiomyocytes. Moreover, SII has cardioprotective effects by diminishing the inflammation, oxidative stress, and apoptosis triggered by Dox. Network pharmacological analysis further shows that SII downregulates P53 protein expression by activating the AKT/MDM2 signaling pathway, thus attenuating DIC. In conclusion, this study confirms that SII mitigates DIC through downregulation of the AKT/MDM2/P53 signaling pathway, suggesting a promising therapeutic strategy for alleviating DIC.

## Introduction

Doxorubicin (Dox) is extensively employed as a chemotherapeutic agent to treat a range of malignancies [1], such as acute leukemia [2], non-Hodgkin’s lymphoma [3], soft tissue sarcoma [4], and breast cancer [5]. Its clinical use has significantly increased cancer patient survival rates, establishing it as a cornerstone of cancer treatment. However, Dox induces various biochemical effects, especially cardiotoxicity, which can culminate in heart failure. Approximately 11% of patients experience Dox-induced cardiotoxicity (DIC) post-treatment, with the incidence being dose dependent: cumulative doses exceeding 400–700 mg/m ^2^ in adults and 300 mg/m ^2^ in children substantially increase the risk of heart failure. Chronic heart failure occurs in approximately 4% of patients receiving 500–550 mg/m ^2^ cumulative doses, increasing to 18% at 600 mg/m ^2^. This severe cardiotoxicity restricts its clinical application. Although the Food and Drug Administration of USA has approved the use of dexrazoxane (Dex) to mitigate DIC [6], its use is associated with complications, including exacerbated myelosuppression, a common adverse effect of chemotherapy [7]. Consequently, the medical community urgently seeks safe and effective therapies to reduce DIC.

*Saussurea involucrata*, a perennial herb from the *Compositae* family, is a rare and esteemed medicinal plant native to Xinjiang [8]. It thrives on the cliffs of the Tianshan Mountains at altitudes approximately 4000 m above sea level. For centuries, this herb has been a vital component of Chinese medicinal practices and, in the past fifty years, has gained broader application owing to its therapeutic properties, health benefits, and culinary uses [9].

According to the Pharmacopoeia of China, *Saussurea involucrata* injection (SII) is a sterile aqueous solution derived from the dried aerial parts of *S*. *involucrata* that has significant anti-inflammatory and anti-oxidative stress effects. For example, *S*. *involucrata* targets key proteins via MAPK- and NF-κB-related pathways to effectively inhibit inflammation and reduce bone destruction in the treatment of rheumatoid arthritis [10]. Moreover, SII modulates the Nrf2/HO-1/NLRP3 pathway to mitigate acute liver injury in mice [11]. Research has also highlighted the cardiovascular protective effects of *S*. *involucrata* .

The cardiotoxic mechanisms of Dox are complex and involve ROS production [12], inflammation promotion [13], apoptosis induction [14], mitochondrial impairment [15], endoplasmic reticulum stress, and autophagy pathway dysregulation [16]. Despite evidence that SII has anti-inflammatory, antioxidant, and cardioprotective properties, no studies have yet documented its potential to mitigate DIC.

Therefore, this study aims to combine network pharmacology with *in vivo* and *in vitro* experiments to validate the efficacy of SII in alleviating DIC and elucidate the underlying mechanisms of its cardioprotective effects.

## Materials and Methods

### Chemicals and reagents

SII was supplied by Xinjiang Pharmaceutical Co., Ltd. (Z65020112; Urumqi, China). Doxorubicin (S17092) and Nutlin3-a (S80888) were obtained from Shanghai Yuanye Biotechnology Co., Ltd. (Shanghai, China). Assay kits for LDH (A02022), CK-MB (E00611), SOD (A00112), GSH-PX (A00512), and MDA (A00312) were obtained from Jiancheng Bioengineering Institute (Nanjing, China). ELISA kits for IL-1β (EK201B), IL-6 (EK206), and TNF-α (EK282) were acquired from MultiSciences Biotech Co., Ltd. (Hangzhou, China). The MTT (ST1537), Nuclear and Cytoplasmic Protein Extraction kit (P0027), DHE (S0063), Enhanced Mitochondrial Membrane Potential Assay kit with JC-1 (C2003S), and Annexin V-FITC Apoptosis Detection kit (C1062L) were purchased from Beyotime Biotechnology (Shanghai, China). The TUNEL Cell Apoptosis Detection kit (G1501), DAPI Staining Solution (G1012), Masson Staining kit (G1006), and Hematoxylin and Eosin Staining kit (G1005) were obtained from SERVICEBIO Technology Co., Limited (Wuhan, China). Dulbecco’s modified Eagle’s medium (DMEM) and fetal bovine serum were obtained from Invitrogen (Carlsbad,, USA). The primary antibodies used in this study included anti-BAX (ab182733, 1:2000; Abcam, Cambridge, USA), anti-BCL-2 (ab194583, 1:500; Abcam), anti-cleaved caspase-3 (ab32042, 1:500; Abcam), anti-P-AKT (ab38449, 1:1000; Abcam), anti-IL-1 beta (ab283818, 1:1000; Abcam), anti-P53 (60283-2-Ig, 1:1000; Proteintech, Wuhan, China), anti-Nrf2 (16396-1-AP, 1:2000; Proteintech), anti-HO-1 (66743-1-Ig, 1:1000; Proteintech), anti-NQO1 (67240-1-Ig, 1:5000; Proteintech), anti-TNF alpha (60291-1-Ig, 1:1000; Proteintech), anti-PCNA (60097-1-Ig, 1:10,000; Proteintech), anti-GAPDH (60004-1-Ig, 1:10,000; Proteintech), anti-phospho-NF-κB p65 (bs-5661R, 1:1000; Bioss, Beijing, China), and anti-IL6 (bs-0782R, 1:1000; Bioss).

### Experimental animals and treatments

C57BL/6 male mice (8 weeks, 22–24 g) were obtained from Sikebeisi Biotechnology Co., Ltd. (Anyang, China). The mice were housed with free access to food and water under a 12/12-h light/dark cycle, with the humidity maintained at 50% ± 5% and the temperature at 20–25°C. All animal experiment procedures were approved by the Animal Protection and Use Committee at Shihezi University (approval number: A2024-004-01) and complied with the university’s ethical guidelines for animal experiments.

The mice were randomly divided into four groups: Ctrl, SII, Dox, and Dox + SII. Starting on day 4, the Dox and Dox + SII groups received Dox injections (5 mg/kg) at five-day intervals, reaching a cumulative dose of 25 mg/kg to induce chronic cardiac injury. Concurrently, from day 1 to day 28, the SII and Dox+SII groups were administered with SII intraperitoneally at a dose of 0.35 mg/kg/day. The Ctrl group received intraperitoneal injections of an equivalent volume of saline solution. Echocardiographic assessments were performed 24 h after the final drug administration. The mice were subsequently anaesthetized with 50 μg/g sodium pentobarbital. Cardiac blood samples were collected, and the hearts were immediately excised. A portion of the cardiac tissue was fixed in 4% paraformaldehyde and embedded in paraffin for sectioning, while the remainder was processed into a homogenate for further analyses. After heart collection, the mice were euthanized via cervical dislocation.

### Cell culture

Rat H9c2 cells (#r012) were obtained from iCell Bioscience, Inc. (Shanghai, China). The cells were cultured in DMEM supplemented with 10% fetal bovine serum and 1% penicillin-streptomycin (Gibco, Carlsbad, USA). H9c2 cells were maintained in a humidified atmosphere with 5% carbon dioxide at 37°C. The cells were divided into six groups: Ctrl, SII, Dox, Nutlin-3a, Dox+SII, and Dox+SII+ Nutlin-3a. Following a 2-h pre-treatment with SII (10 μg/mL), the cells were exposed to Dox (1 μM) for 24 h. To stabilize P53, 10 μM Nutlin-3a was added to the Nutlin-3a group and Dox+SII+Nutlin-3a group.

### MTT assay

H9c2 cells were seeded in 96-well plates at a density of 8000 cells per well. The SII and Dox + SII groups were pretreated with various concentrations of SII (0.01, 0.1, 1, 10, or 100 μg/mL) for 2 h. The Dox and Dox+SII groups were subsequently treated with Dox at concentrations of 0.25, 0.5, 1, 2, and 4 μM for 24 h. After the treatment period, the cell culture medium was removed, and 0.5 mg/mL MTT solution was added to each well, followed by incubation at 37°C for 3 h. The MTT solution was then discarded, and 150 μL of DMSO was added to each well. The absorbance was measured at 490 nm using a microplate reader (Thermo Fisher Scientific, Waltham, USA).

### Biochemical analysis and enzyme activity detection

A commercial kit from MultiSciences Biotech (Nanjing, China) was utilized, following the manufacturer’s instructions, to measure the levels of myocardial enzymes (CK-MB and LDH) and indicators of oxidative stress (MDA, GSH-Px, and SOD) in heart tissues or cell medium.

### ELISA

The levels of TNF-α, IL-1β, and IL-6 in mouse cardiac tissue homogenates were measured via commercially available ELISA kits in accordance with the manufacturer’s guidelines. In brief, after the mouse heart tissue homogenate was mixed and reacted with the corresponding reagent, the optical density values at 450 nm were detected through enzyme standardization, and the standard curve was plotted. The measurements were repeated six times for both standards and samples.

### Western blot analysis

Total proteins were isolated from mouse heart tissues and H9c2 cells using RIPA lysis buffer supplemented with 1% PMSF and 1% protein phosphatase inhibitor. Protein quantification was performed using a BCA protein assay kit (Thermo Fisher Scientific). Proteins were then separated on sodium dodecyl sulfate-polyacrylamide gels and transferred onto PVDF membranes (Millipore, Billerica, USA). The membranes were blocked with 5% skim milk for 2 h at room temperature and then incubated overnight with primary antibodies at 4°C. Following primary antibody incubation, the membranes were incubated with secondary antibodies (Proteintech, Wuhan, China) at a dilution of 1:10,000 for 2 h at room temperature. Detection involved incubating the membranes with chemiluminescence reagents from the ECL kit (Biosharp, Beijing, China) and capturing the emitted light using a chemiluminescence imager. The resulting images were quantitatively analyzed with ImageJ software (National Institutes of Health, Bethesda, USA).

### Flow cytometry assay

The treated cells were loaded with fluorescent probes and subsequently washed with PBS to remove any excess. The resulting cell suspension was analyzed using a flow cytometer (BD Biosciences, Franklin Lakes, USA). Intracellular ROS levels were measured using DHE staining, apoptosis was assessed via Annexin V/PI staining, and the mitochondrial membrane potential (MMP) was determined through JC-1 staining. The intracellular ROS levels, MMP, and apoptosis rates were quantitatively analyzed using FlowJo V10 software (TreeStar, Ashland, USA).

### Echocardiography

The mice were anaesthetized with 1% isoflurane in oxygen 24 h after the final dose and examined by echocardiography using the Vevo 3100 Microultrasound imaging system (Visual Sonics Inc., Toronto, Canada). Cardiac function indices were analyzed with Vevo LAB 3.3.1 software (Fujifilm Visual Sonics, Toronto, Canada). All indicators were evaluated over three sequential cardiac cycles.

### DHE staining

The level of ROS in cardiac tissue was detected using DHE staining. Frozen sections of mouse hearts were stained with a 5 μM DHE dye solution and incubated at 37°C in a dark environment for 30 min. Images were then captured using a fluorescence microscope (Nikon, Tokyo, Japan). Quantitative analysis was performed using ImageJ software.

### TUNEL staining

Following the manufacturer’s guidelines, TUNEL/DAPI double staining was performed on paraffin sections of mouse hearts to detect cardiomyocyte apoptosis. The paraffin sections were first dewaxed using xylene. Next, the samples were incubated with proteinase K working solution at 37°C for 15 min. The sections were then incubated with the TUNEL working solution at 37°C in a dark environment for 60 min. After washing with PBS, the nuclei were stained with DAPI. Finally, images were captured using a fluorescence microscope (Nikon) and quantitatively analyzed with ImageJ software. The cell apoptosis rate was calculated as follows: cell apoptosis rate = (the number of TUNEL-positive cells/total cells) × 100%.

### Histological staining

Following euthanasia, the mouse hearts were perfused with normal saline, followed by a 4% paraformaldehyde solution. The hearts were then removed and placed in a 4% paraformaldehyde solution overnight. The hearts were subsequently sliced into 5-μm-thick sections and embedded in paraffin. Histological morphology was observed using H&E staining of the paraffin sections. Myocardial collagen deposition was detected via Masson staining of the paraffin sections. The stained sections were then observed and photographed using a light microscope, facilitating the examination and analysis of histological features and collagen deposition in the mouse hearts.

### Network pharmacology analysis

In this study, the TCMSP database ( http://tcmspw.com/tcmsp.php) was used to identify the active ingredients of *S*. *involucrata*, with their structural formulas obtained from the PubChem website ( https://pubchem.ncbi.nlm.nih.gov/). The target genes of these active ingredients were predicted using the TCMSP database, Swiss database ( www.swisstargetprediction.ch), and TargetNet database ( targetnet.scbdd.com/home/index). Differentially expressed genes (DEGs) from the GEO database ( https://www.ncbi.nlm.nih.gov/geo/) were compared in human multifunctional stem cell-induced cardiomyocytes (GSE217423) with and without Dox intervention. DEGs were cross-referenced with the predicted targets of active ingredients using a Venn diagram, and common targets were identified. These common targets were input into the STING database ( https://string-db.org/) to construct a protein-protein interaction network. The common targets were ranked on the basis of the parameters of protein interactions and analyzed using Cytoscape 3.9.1 software. Gene Ontology (GO) and Kyoto Encyclopedia of Genes and Genomes (KEGG) enrichment analyses of the common targets were performed via the David database ( https://david.ncifcrf.gov/summary.jsp) and the OmicShare website ( https://www.omicshar.com/), and dynamic enrichment bubble plots of GO analysis and signaling pathways were generated.

### Statistical analysis

**Figure 5:**
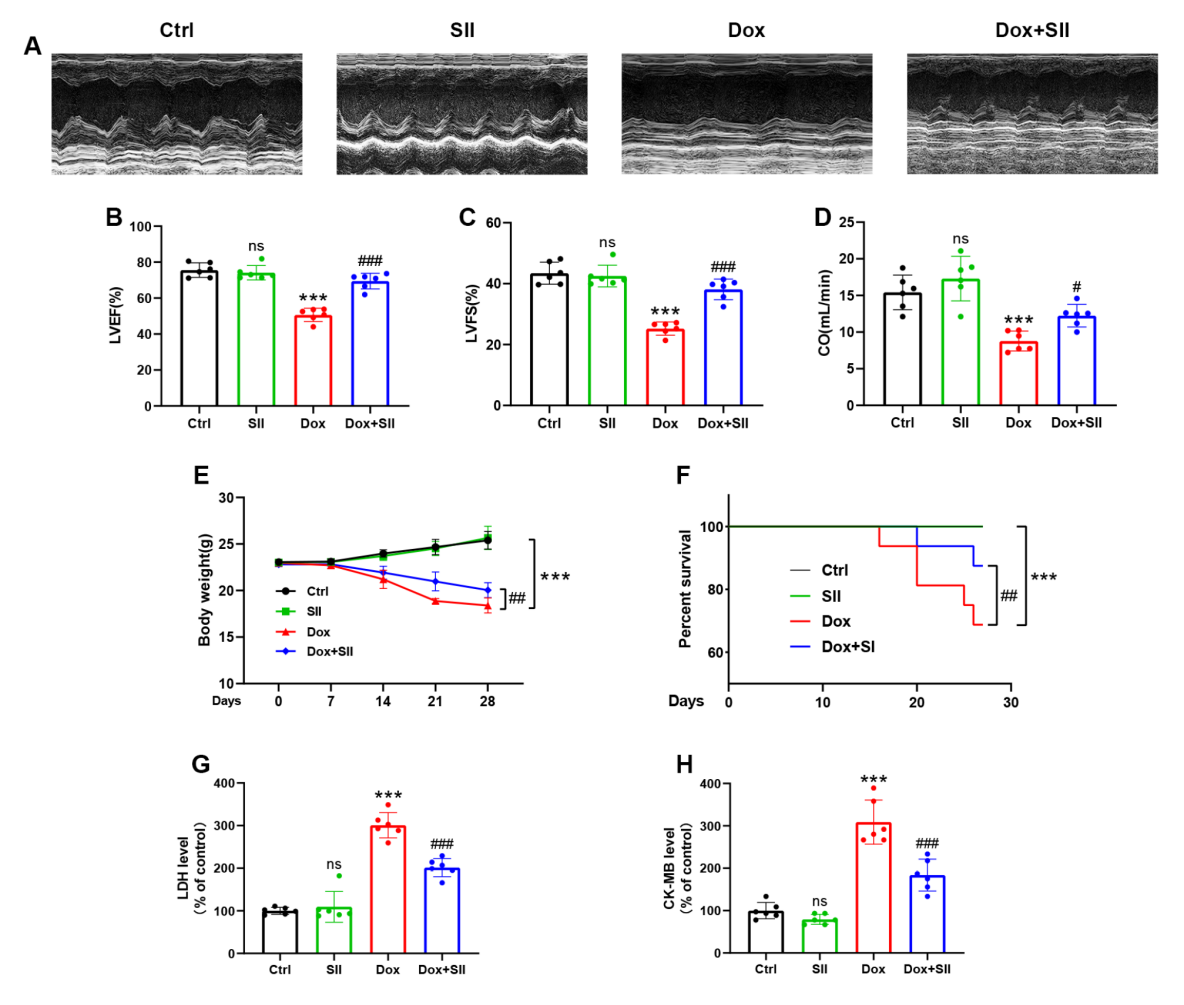
Figure 5 SII attenuates DIC *in vivo* (A) Cardiac function of mice in each group evaluated by echocardiography. (B–D) Quantitative analysis of (B) LVEF, (C) LVFS, and (D) CO. (E) Body weight of the mice monitored and recorded every 7 days. (F) Kaplan-Meier survival curves for each group. (G,H) Heart tissue levels of LDH (G) and CK-MB (H) in mice. Data are presented as the mean ± SD, n = 6. ns, not significant. *P < 0.05, **P < 0.01, ***P < 0.001 vs Ctrl group; #P < 0.05, ##P < 0.01, ###P < 0.001 vs Dox group. A two-way ANOVA analysis was used in Figure 5E; The Log-rank (Mantel-Cox) test was used in Figure 5F; One-way analysis of variance was used for others.

IBM SPSS Statistics 26 software was used for statistical analysis of the experimental data. Two-way ANOVA was used in Figure 5E; the log-rank (Mantel-Cox) test was used in Figure 5F; and one-way analysis of variance was used for other comparisons. Data are presented as the mean ± standard deviation (SD), and corresponding statistical charts were generated on the basis of these results. A *P* value of less than 0.05 was considered statistically significant.

## Results

### SII attenuates Dox-induced injury *in vitro*

**Figure 1:**
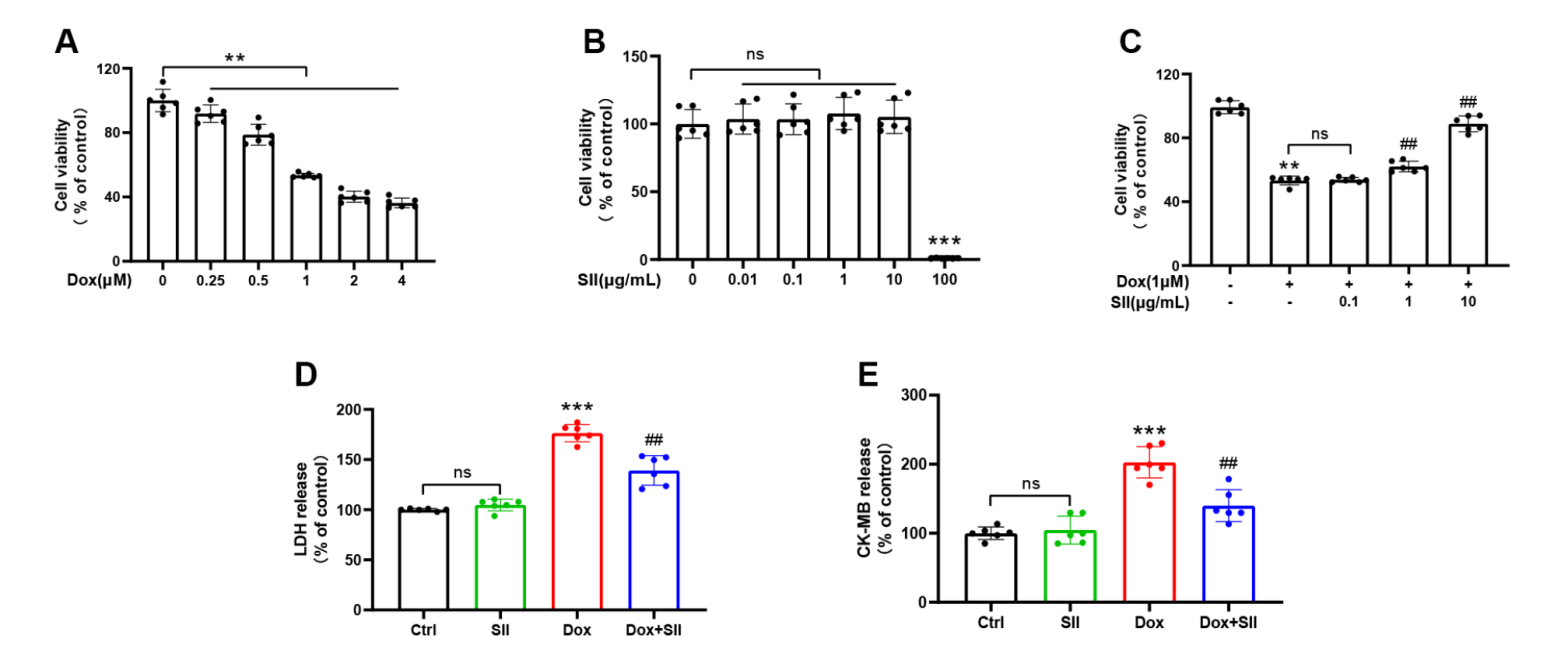
Figure 1 SII attenuates Dox-induced injury in H9c2 cells (A) Effect of Dox on H9c2 cell viability. (B) Effects of SII on H9c2 cell viability. (C) Effect of different SII concentrations on the viability of Dox-treated H9c2 cells. Effect of SII on LDH (D) and CK-MB (E) levels in Dox-treated H9c2 cells. Data are presented as the mean ± SD, n = 6. ns, not significant. ** P < 0.01, ***P < 0.001 vs Ctrl group; ## P < 0.01 vs Dox group.

MTT assays were used to assess cell viability to investigate the impact of SII on DIC in H9c2 cells. The data in Figure 1A showed a significant concentration-dependent reduction in cell viability following Dox administration at doses of 0.25, 0.5, 1, 2, and 4 μM over 24 h. Figure 1B revealed that SII at concentrations of 0.01, 0.1, 1, and 10 μg/mL did not affect H9c2 cell viability during the same period. Remarkably, as shown in Figure 1C, co-treatment with SII at 1 and 10 μg/mL significantly increased cell viability compared with that of cells treated with only 1 μM Dox or with 0.1 μg/mL SII and 1 μM Dox. Enzyme activity assays revealed elevated LDH and CK-MB levels in cell medium supernatants after Dox treatment, whereas co-treatment with SII markedly reduced Dox-induced LDH and CK-MB release ( Figure 1D,E). These findings suggested that SII can attenuate Dox-induced injury *in vitro*.

### SII attenuates Dox-induced inflammation *in vitro*

**Figure 2:**
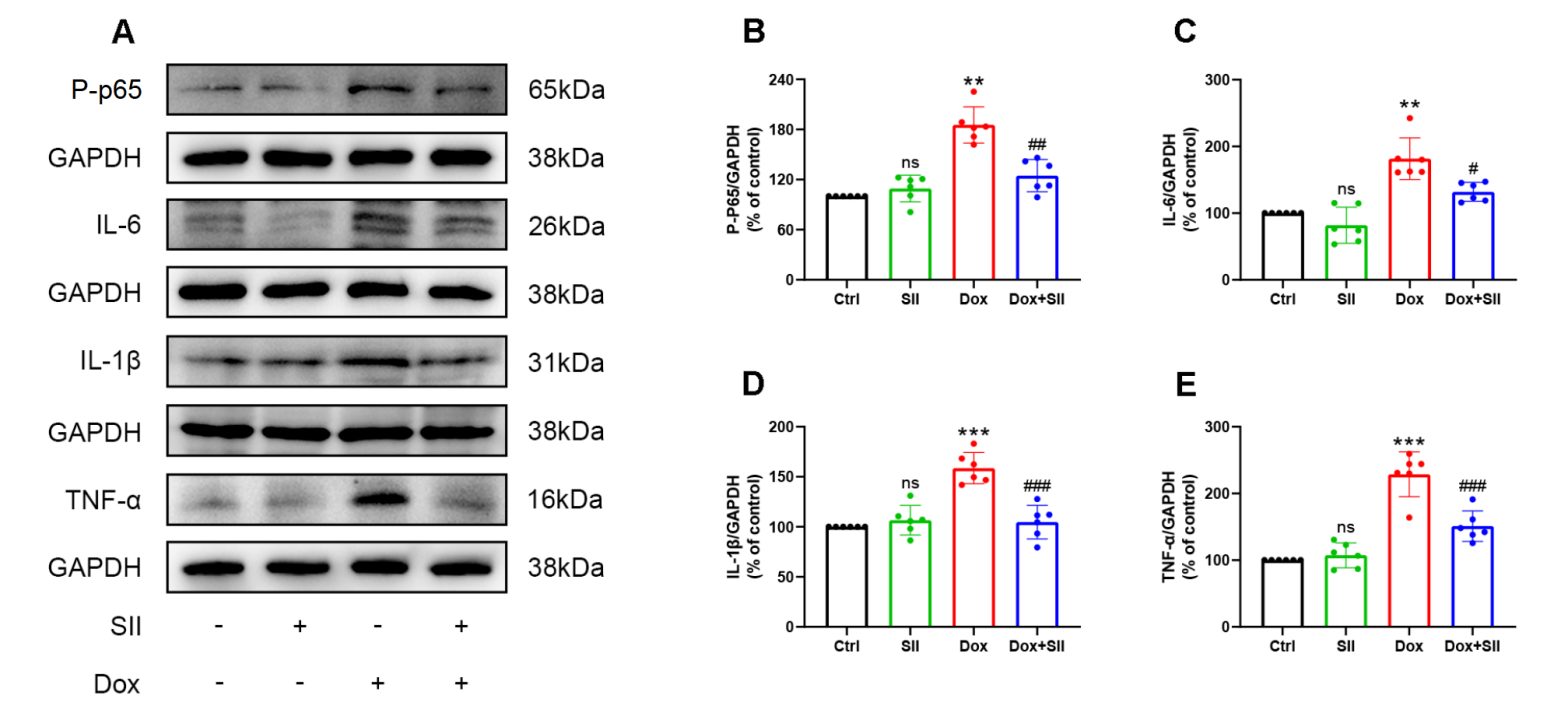
Figure 2 SII attenuates Dox-induced inflammation *in vitro* (A) Effect of SII on Dox-induced P-p65 and inflammatory factors (IL-6, IL-1β, and TNF-α) expressions in H9c2 cells examined by western blot analysis. Statistical analysis of protein expression levels for P-p65 (B), IL-6 (C), IL-1β (D), and TNF-α (E). Data are presented as the mean ± SD, n = 6. ns, not significant. ** P < 0.01, ***P < 0.001 vs Ctrl group; # P < 0.05, ##P < 0.01, ### P < 0.001 vs Dox group.

To evaluate the anti-inflammatory effects of SII on Dox-treated H9c2 cells, the expression levels of phosphorylated nuclear factor-kappaB P65 (P-p65) and the inflammatory cytokines IL-6, IL-1β, and TNF-α were measured using western blot analysis ( Figure 2A‒E). Compared with those in the Ctrl group, the expression levels of P-p65, IL-6, IL-1β, and TNF-α in cells treated with Dox were significantly increased. However, concurrent treatment with Dox and SII resulted in markedly lower levels of these inflammatory proteins in H9c2 cells than in cells treated with Dox alone. These findings indicated that SII can attenuate Dox-induced inflammation *in vitro*.

### SII attenuates Dox-induced oxidative stress *in vitro*

**Figure 3:**
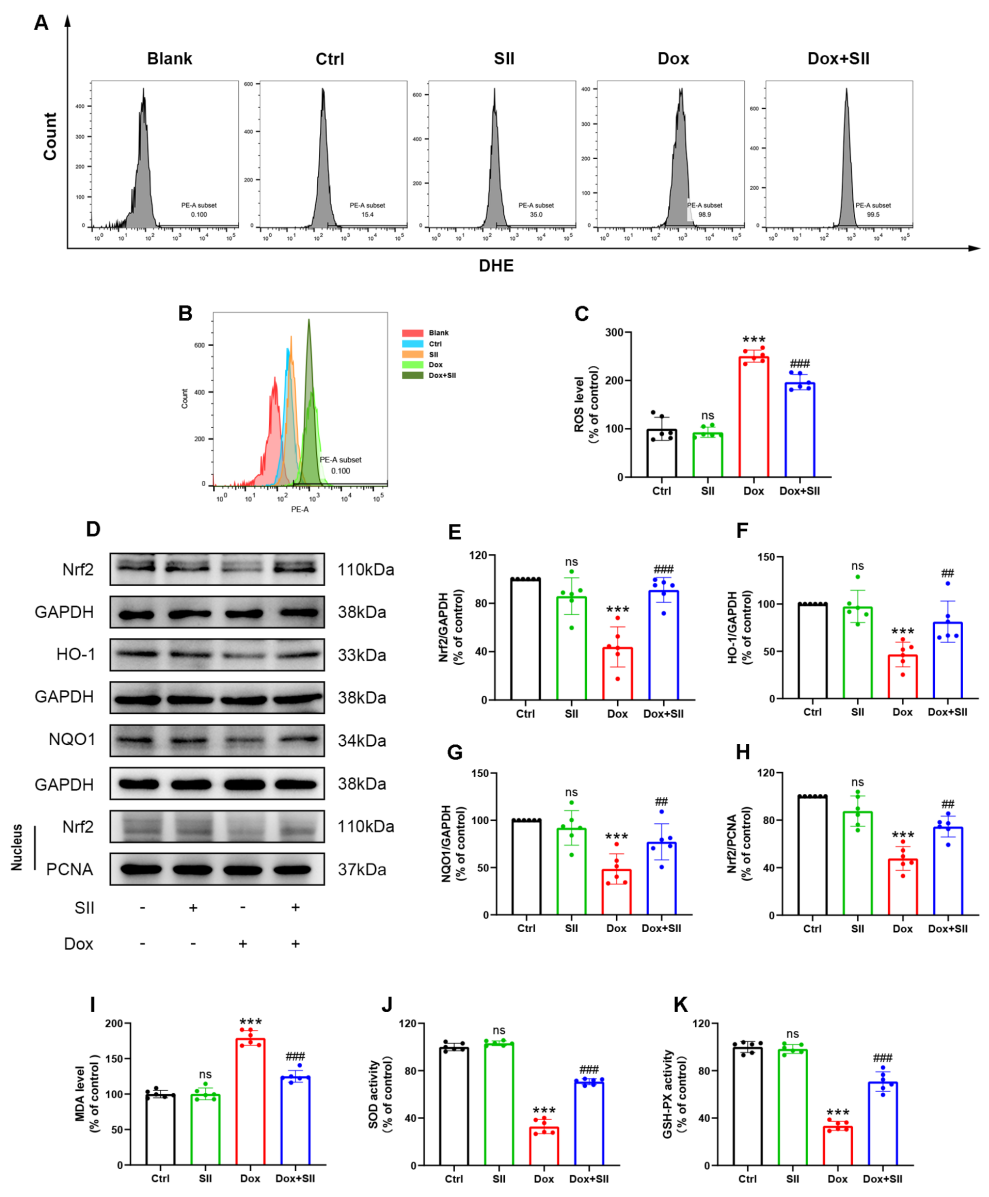
Figure 3 SII attenuates Dox-induced oxidative stress *in vitro* (A,B) Effects of SII on Dox-induced ROS levels in H9c2 cells detected by flow cytometry. (C) Statistical results of flow cytometry. (D–H) Effects of SII on Dox-induced Nrf2, HO-1, and NQO1 protein expressions in H9c2 cells examined by western blot analysis. (I–K) Effect of SII on the level of MDA (I) and the activities of SOD (J) and GSH-Px (K) in Dox-treated H9c2 cells. Data are presented as the mean ± SD, n = 6. ns, not significant. ***P < 0.001 vs Ctrl group; ## P < 0.01, ###P < 0.001 vs Dox group.

To investigate the effect of SII on Dox-induced oxidative stress in H9c2 cells, intracellular ROS levels were assessed using DHE staining and flow cytometry analysis. The results showed that SII mitigated the increase in ROS levels caused by Dox in H9c2 cells ( Figure 3A‒C). Additionally, the expression of Nrf2 and its downstream antioxidant proteins HO-1 and NQO1 was evaluated by western blot analysis. Compared with control H9c2 cells, Dox-treated H9c2 cells presented significant reductions in Nrf2, HO-1, and NQO1 expression levels. In contrast, SII treatment notably prevented these adverse effects induced by Dox ( Figure 3D‒H). Furthermore, biochemical analysis was used to measure the levels of MDA and the activities of the antioxidant enzymes SOD and GSH-Px. The findings revealed an increase in MDA in the Dox-treated group, accompanied by decreased activities of SOD and GSH-Px. However, co-treatment with SII had an inhibitory effect on Dox-induced oxidative damage ( Figure 3I‒K). In conclusion, these data suggested that SII can effectively attenuate Dox-induced oxidative stress *in vitro*.

### SII attenuates Dox-induced cardiomyocyte apoptosis *in vitro*

**Figure 4:**
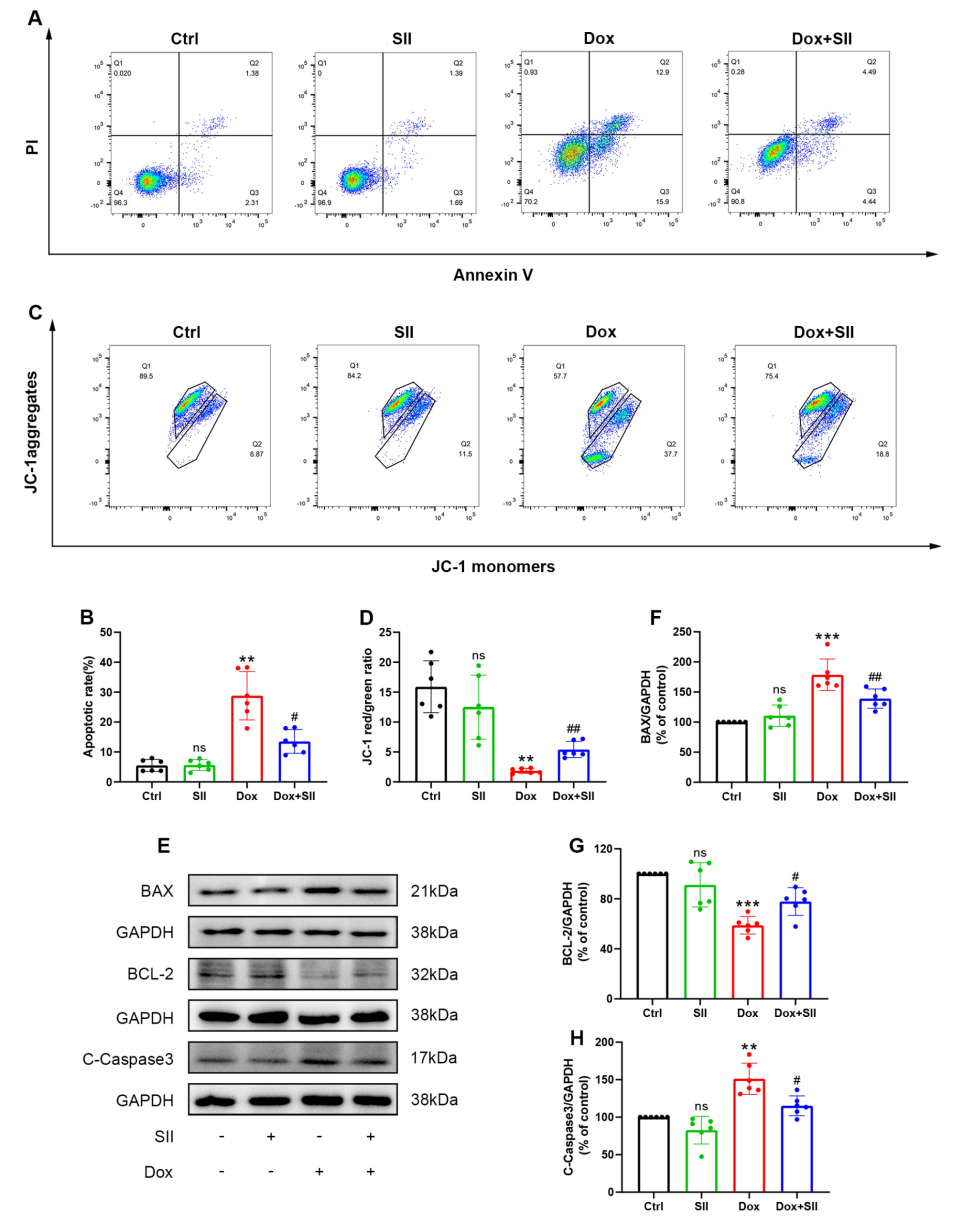
Figure 4 SII attenuates Dox-induced cardiomyocyte apoptosis *in vitro* (A,B) Effects of SII on Dox-induced apoptosis of H9c2 cells detected by flow cytometry. (C,D) Flow cytometry was used to estimate MMP in each group. (E–H) Effects of SII on Dox-induced BAX, BCL-2, and cleaved caspase-3 protein expressions in H9c2 cells examined by western blot analysis. Data are presented as the mean ± SD, n = 6. ns, not significant. **P < 0.01, ***P < 0.001 vs Ctrl group; #P < 0.05, ##P < 0.01 vs Dox group.

Flow cytometry with Annexin V/PI double staining was used to quantify the percentage of apoptotic cells ( Figure 4A), whereas JC-1 staining was used to assess the MMP ( Figure 4B). Compared with control H9c2 cells, Dox-treated H9c2 cells presented a significantly higher apoptosis rate. Notably, co-treatment with Dox and SII significantly reduced apoptosis, indicating that SII has a protective effect against Dox-induced apoptosis ( Figure 4C). Furthermore, SII prevented the Dox-induced decrease in the MMP ( Figure 4D). Western blot analysis revealed the expression levels of the apoptosis-related proteins BAX, BCL-2, and cleaved caspase-3. SII treatment inhibited Dox-induced overexpression of BAX and cleaved caspase-3 and prevented the downregulation of BCL-2 ( Figure 4E‒H). These findings suggested that SII effectively mitigates Dox-induced apoptosis *in vitro* .

### SII attenuates DIC *in vivo*

The echocardiography results revealed significant reductions in Cardiac Output (CO), Left Ventricular Ejection Fraction (LVEF), and Left Ventricular Fractional Shortening (LVFS) in the Dox group compared with those in the Ctrl group. However, SII co-treatment significantly ameliorated Dox-induced cardiac dysfunction ( Figure 5A‒D). Additionally, SII inhibited the concomitant weight loss and reduced the survival rates induced by Dox ( Figure 5E,F). Analysis of LDH and CK-MB levels in heart tissues revealed that SII effectively curbed the Dox-induced increase in these biomarkers ( Figure 5G,H). These findings suggest that SII provides protective effects against DIC *in vivo*.

### SII attenuates Dox-induced cardiomyocyte injury *in vivo*

**Figure 6:**
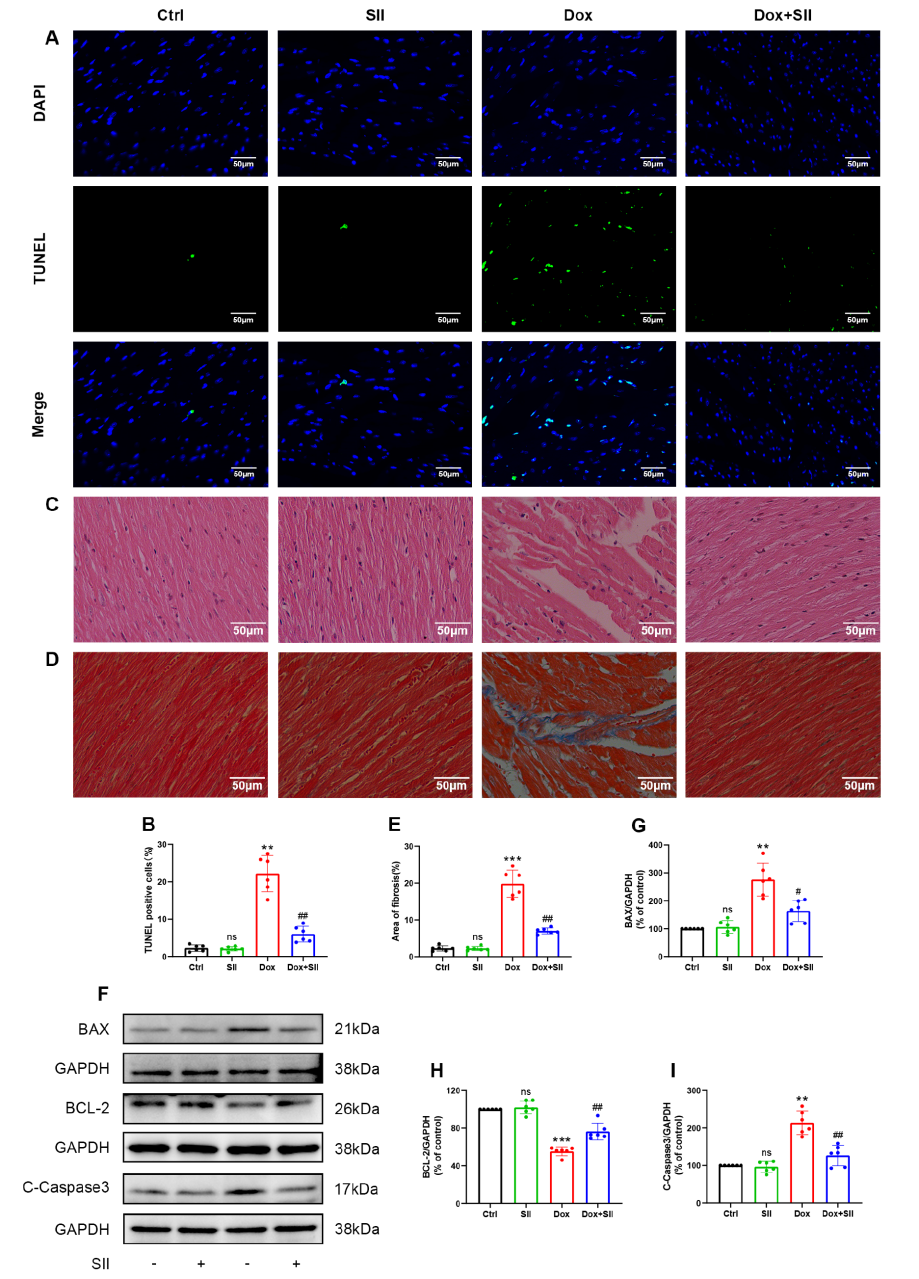
Figure 6 SII attenuates Dox-induced cardiomyocyte injury *in vivo* (A,B) Effect of SII on Dox-induced myocardial apoptosis in mice observed by TUNEL staining and its statistical result. (C) Effect of SII on Dox-induced pathological changes in myocardial tissues in mice observed by H&E staining. (D,E) Effect of SII on Dox-induced myocardial fibrosis in mice and the statistical result of fibrotic area. (F–I) Effect of SII on Dox-induced BAX, BCL-2, and C-caspase-3 protein expressions in mouse hearts examined by western blot analysis. Data are presented as the mean ± SD, n = 6. ns, not significant. ** P < 0.01, ***P < 0.001 vs Ctrl group; # P < 0.05, ##P < 0.01 vs Dox group.

To investigate the impact of SII on Dox-induced cardiomyocyte damage in mice, TUNEL staining was used to assess apoptosis. Compared with the Ctrl group, the Dox group exhibited a significantly increased rate of cardiomyocyte apoptosis, which was mitigated by SII injection ( Figure 6A,B). Additionally, H&E staining revealed that SII ameliorated pathological changes such as swelling, degeneration of cardiomyocytes, and muscle fiber rupture caused by Dox in mice ( Figure 6C). Masson′s trichrome staining revealed that SII also reduced Dox-induced fibrosis in cardiomyocytes ( Figure 6D,E). Western blot analysis corroborated these findings, revealing the increased expressions of C-caspase-3 and BAX and the decreased expression of BCL-2 in the Dox group, effects that were counteracted by SII treatment ( Figure 6F–I). These results suggested that SII has a cardioprotective effect against Dox-induced damage in mouse cardiomyocytes.

### SII attenuates Dox-induced inflammation and oxidative stress *in vivo*

**Figure 7:**
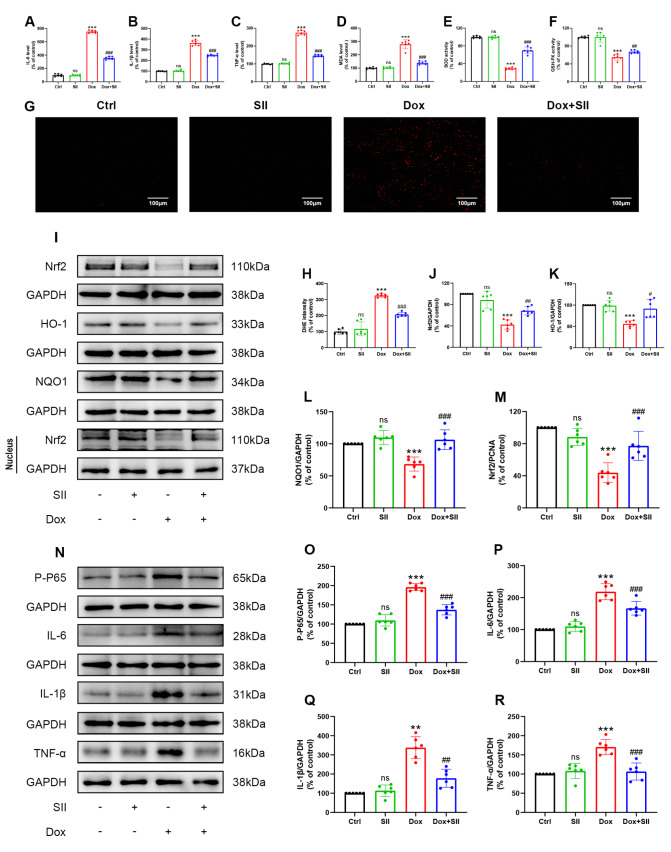
Figure 7 SII reduces Dox-induced inflammation and oxidative stress *in vivo* (A–C) Effect of SII on the levels of IL-6, IL-1β, and TNF-α induced by Dox, determined by ELISA in mouse hearts. (D–F) Effect of SII on the level of MDA and the activities of SOD and GSH-Px in mouse hearts induced by Dox. (G,H) Effects of SII on Dox-induced ROS levels in mouse hearts detected by DHE staining. (I–M) Effect of SII on Dox-induced Nrf2, HO-1, and NQO1 protein expressions in mouse hearts examined by western blot analysis. (N–R) Effect of SII on Dox-induced P-p65 and inflammatory factors (IL-6, IL-1β, and TNF-α) expressions in mouse hearts examined by western blot analysis. Data are presented as the mean ± SD, n = 6. ns, not significant. **P < 0.01, ***P < 0.001 vs Ctrl group; #P < 0.05, ##P < 0.01, ###P < 0.001 vs Dox group.

To determine the ability of SII to ameliorate Dox-induced inflammation and oxidative stress in mouse hearts, ELISA was used to measure the expression levels of the inflammatory cytokines IL-6, IL-1β, and TNF-α in heart tissue homogenates. The results revealed a significant increase in these cytokines following Dox treatment, which was notably decreased by SII ( Figure 7A‒C). Additionally, MDA levels and the activities of SOD and GSH-Px were assessed in mouse heart tissues. SII prevented the significant decrease in SOD and GSH-Px activity and the increase in MDA levels induced by Dox ( Figure 7D‒F). DHE staining via fluorescence microscopy was used to assess ROS levels in frozen heart sections. The findings revealed a substantial increase in ROS in Dox-treated hearts, whereas SII treatment decreased ROS levels ( Figure 7G,H). The results of the western blot analysis supported these findings, which aligns with the *in vitro* findings. SII mitigated the Dox-induced upregulation of P-p65, IL-6, IL-1β, and TNF-α ( Figure 7N‒R) and counteracted the downregulation of Nrf2, HO-1, and NQO1 ( Figure 7I‒M). These findings suggested that SII effectively reduces Dox-induced inflammation and oxidative stress *in vivo*.

### Network pharmacology analysis

**Figure 8:**
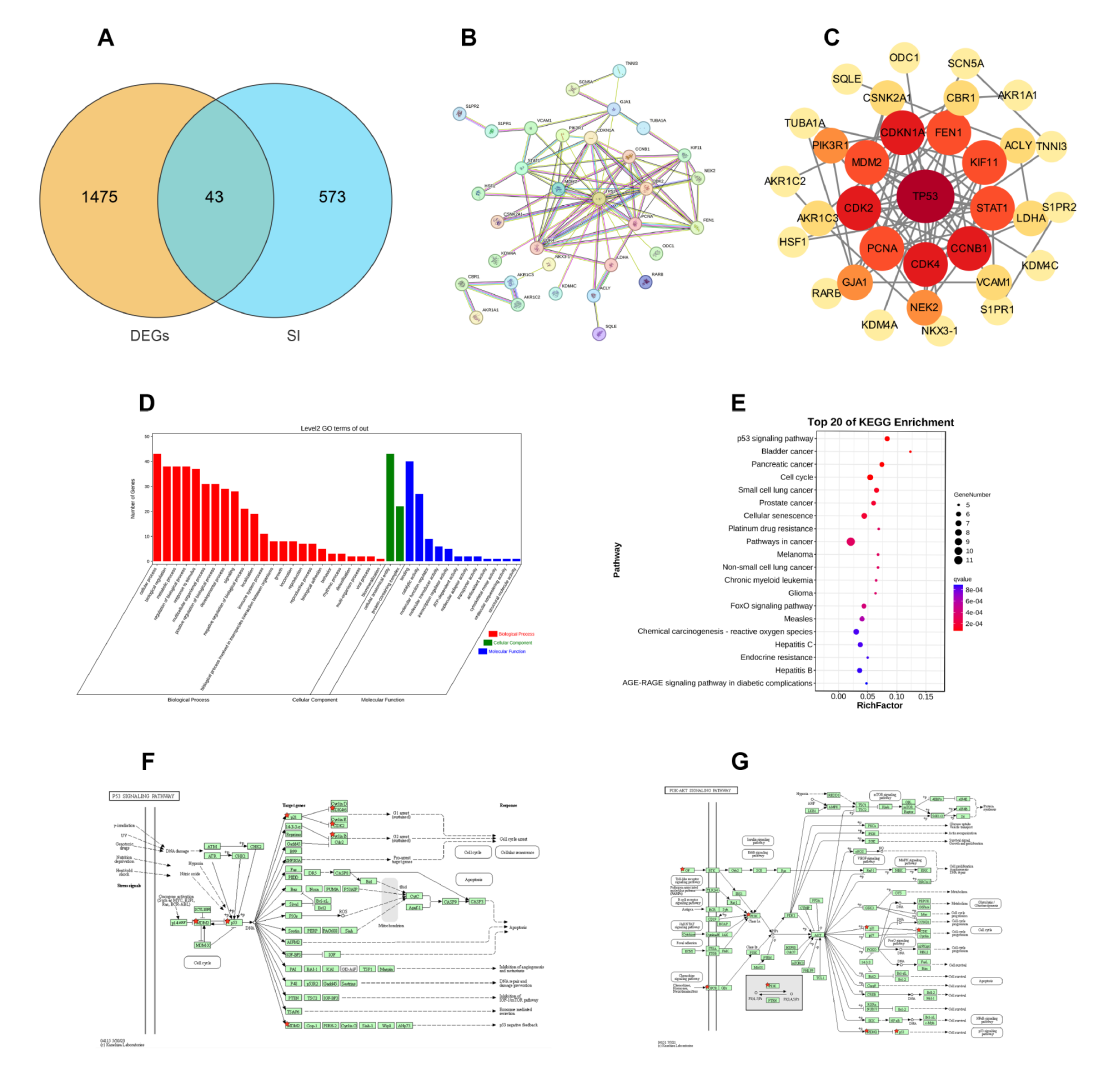
Figure 8 Results of network pharmacology analysis (A) Venn diagram of the common targets of SI-DEGs. (B) Common target protein interaction network of SI-DEGs. (C) Ranking of common targets for SI-DEGs. (D) GO enrichment analysis of the 43 common targets. (E) KEGG enrichment analysis of the 43 common targets. (F,G) The 43 common target genes enriched in the AKT/MDM2/P53 signaling pathway.

By applying the criteria of oral bioavailability (OB) ≥30% and drug-likeness (DL) ≥0.18, seven active compounds within *S*. *involucrata* were identified using the TCMSP database, including dinatin, alloisoimperatorin, beta-sitosterol, kaempferol, luteolin, flazin, and quercetin, along with their corresponding targets. The structural formulas of these compounds were retrieved from PubChem. Using these structures, the Swiss and TargetNet databases were used to predict additional targets. Amalgamating the predicted targets from the TCMSP, Swiss, and TargetNet databases yielded 616 unique targets postduplication. Moreover, the GEO database revealed 1518 genes differentially expressed in cardiomyocytes following Dox treatment compared with those in the control group. By cross-referencing these genes with the 616 potential targets, 43 common targets were identified, as visualized in a Venn diagram ( Figure 8A). Network analysis of these targets *via* the STRING database enabled the creation of degree and protein-protein interaction (PPI) networks ( Figure 8B). Cytoscape 3.9.1 software further refined these data, producing a PPI network ranked by degree value ( Figure 8C). Closer examination revealed the top ten core targets: TP53, CDK4, CDK2, CCNB1, CDKN1A, STAT1, MDM2, PCNA, FEN1, and KIF11.

### GO and KEGG enrichment analyses

GO enrichment analysis revealed that the shared targets primarily regulate cellular processes and respond to biological stimuli ( Figure 8D). KEGG enrichment analysis revealed a significant association between the 43 common targets and the P53 signaling pathway ( Figure 8E). This association implies that the effectiveness of *S*. *involucrata* in treating DIC may involve the P53 signaling pathway. Furthermore, pathway enrichment analysis using the DAVID database suggested that SII may mitigate Dox-induced cardiotoxic effects through modulation of the AKT/MDM2/P53 axis ( Figure 8F). Within this context, P53 and MDM2 are identified as the primary and seventh core targets, respectively.

### Effects of SII on AKT, MDM2, and P53 protein expression in DIC models *in vitro* and *in vivo*

**Figure 9:**
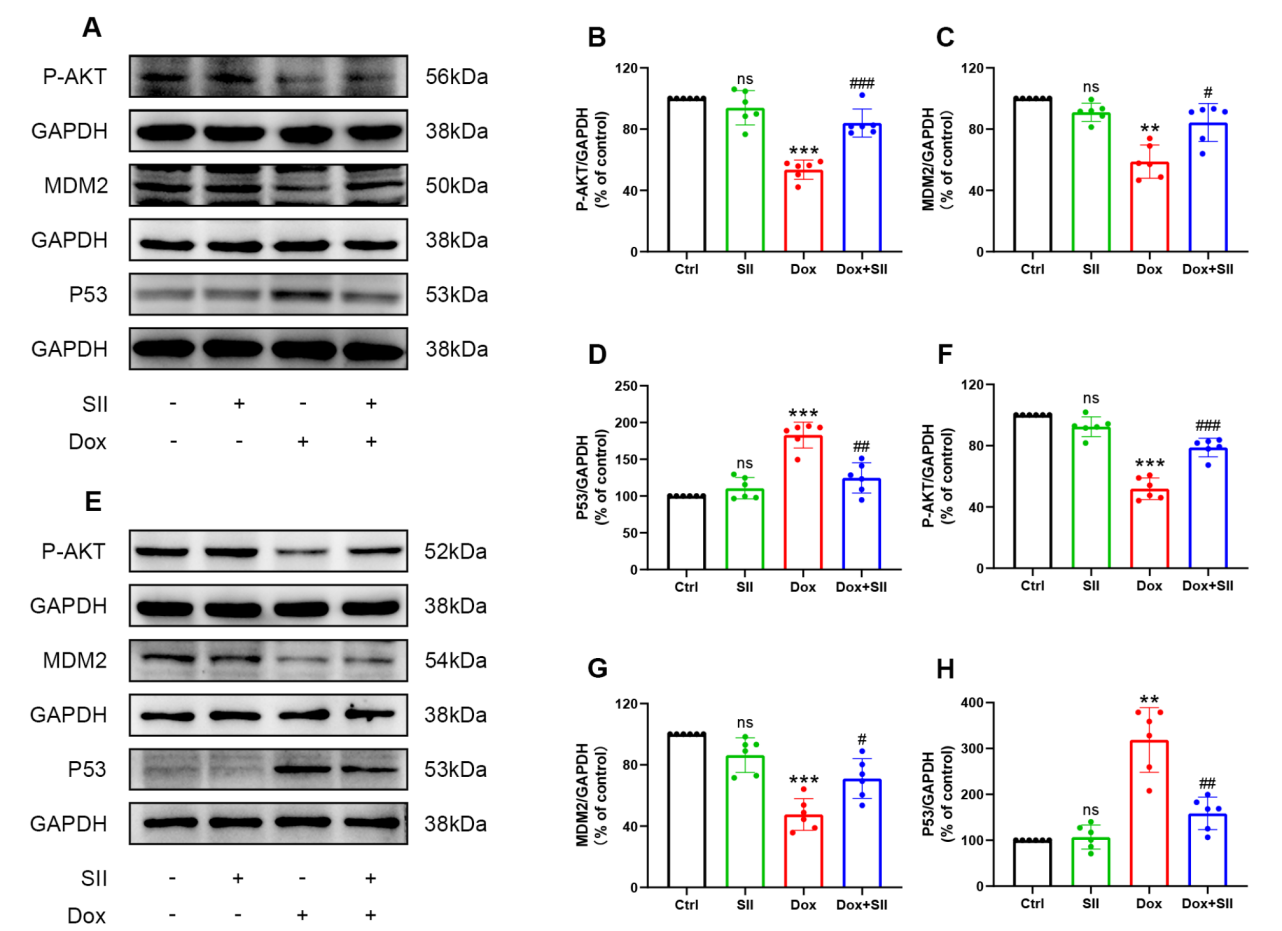
Figure 9 Effects of SII on AKT, MDM2, and P53 protein expressions in DIC models *in vitro* and *in vivo* Effects of SII on AKT, MDM2, and P53 protein expressions in DIC models in vivo (A–D) and in vitro (E–H). Data are presented as the mean ± SD, n = 6. ns, not significant. **P < 0.01, *** P < 0.001 vs Ctrl group; #P < 0.05, ##P < 0.01, ###P < 0.001 vs Dox group.

Protein extracts from mouse heart tissues and rat H9c2 cells were analyzed to evaluate the protein expression levels of phosphorylated AKT, MDM2, and p53 through western blot analysis ( Figure 9A,E). Compared with Ctrl treatment, Dox treatment significantly suppressed phosphorylated AKT and MDM2 protein levels while increasing P53 protein expression. Notably, SII treatment prevented these effects of Dox ( Figure 9B‒D,F‒H). In conclusion, the ability of SII to prevent Dox-induced cardiomyocyte damage is attributed to its regulatory influence on the AKT/MDM2/P53 axis.

### SII ameliorates DIC by downregulating the AKT/MDM2/P53 signaling pathway

**Figure 10:**
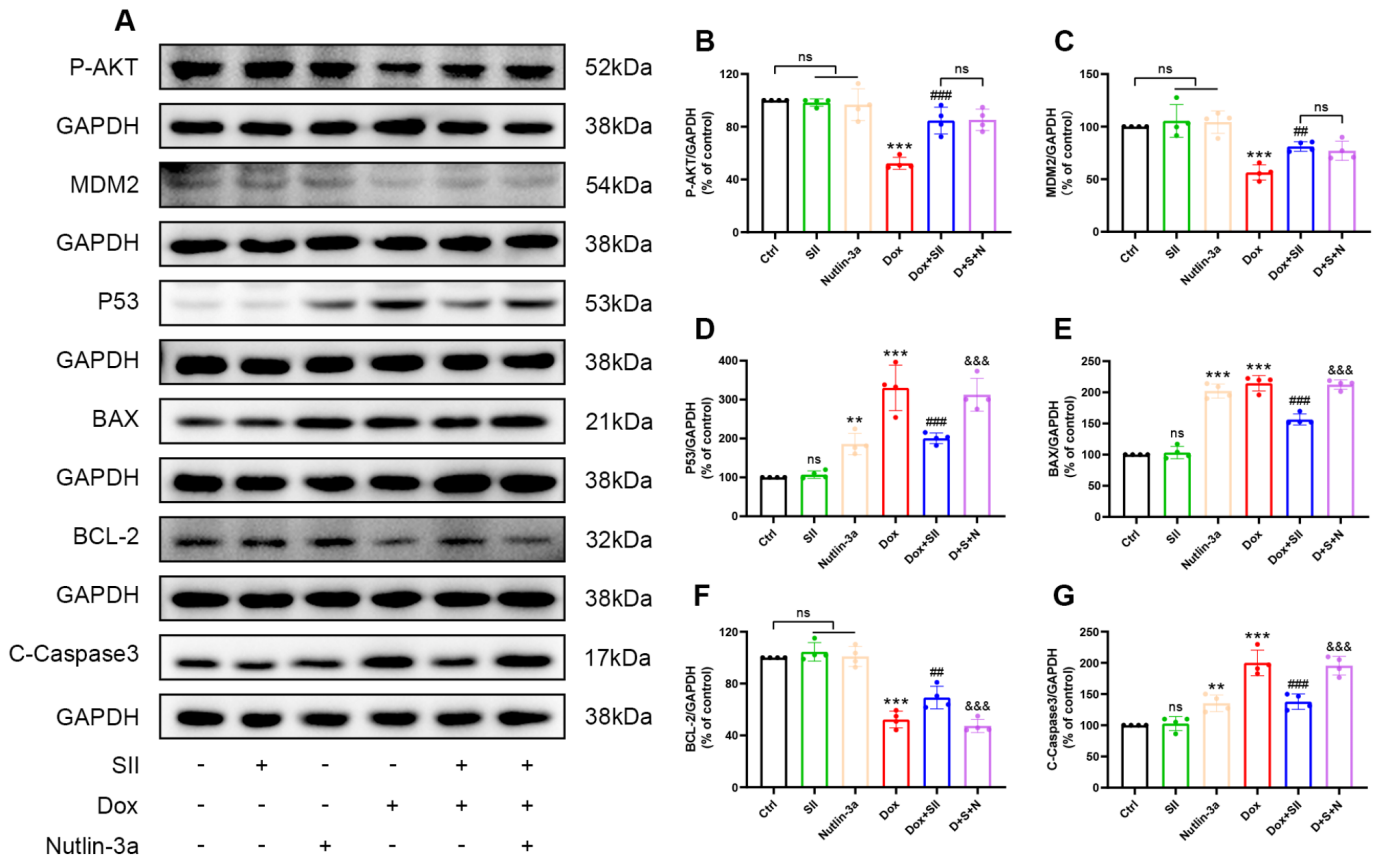
Figure 10 SII ameliorates DIC by downregulating the AKT/MDM2/P53 signaling pathway (A) Western blot analysis of P-AKT, MDM2, P53, BAX, BCL-2, and C-caspase3 proteins in H9c2 cells. (B–E) Statistical analysis of protein expression levels of P-AKT, MDM2, P53, BAX, BCL-2, and C-caspase3. Data are presented as the mean ± SD, n = 4. ns, not significant. **P < 0.01, ***P < 0.001 vs Ctrl group; ##P < 0.01, ### P < 0.001 vs Dox group; &&&P < 0.001 vs Dox + SII group.

Nutlin-3a, an inhibitor of MDM2 and activator of P53, stabilizes P53 by inhibiting its interaction with MDM2, reducing P53 degradation. To further validate the mechanism, the experimental design was expanded to include a P53 agonist Nutlin-3a group and a Dox + SII + Nutlin-3a (D + S + N) group. The specific groups were Ctrl, SII, Nutlin-3a, Dox, Dox + SII, and D + S + N. The protein levels of P-AKT, MDM2, P53, BAX, C-caspase3, and BCL-2 were quantified by western blot analysis. As shown in Figure 10, compared with the Dox+SII group, the D + S + N group presented significantly increased levels of P53, BAX, and C-caspase3 proteins and a significantly decreased level of BCL-2 protein in H9c2 cells, whereas the protein levels of P-AKT and MDM2 remained unchanged. These findings indicate that Nutlin-3a pretreatment inhibits P53 degradation by MDM2 and prevents SII-mediated P53 downregulation through the AKT/MDM2/P53 signaling pathway, negating the cardioprotective effects of SII against Dox-induced toxicity. In conclusion, the ameliorative effect of SII on DIC is mediated by downregulation of the AKT/MDM2/P53 signaling pathway.

## Discussion

Dox administration during chemotherapy leads to various adverse reactions, particularly cardiotoxicity, which increases mortality risk among patients with cancer and limits its widespread use [17]. Dox-induced cardiac damage involves inflammatory responses, oxidative stress, autophagy, apoptosis, pyroptosis, and mitochondrial damage. Targeting these pathological processes may help reduce cardiotoxicity. *Saussurea involucrata* has been demonstrated to have anti-inflammatory, antioxidant, and cardiovascular protective effects, suggesting its potential in mitigating DIC.

The results of the present study indicated that Dox treatment significantly diminished cardiac function in mice, as evidenced by a reduced LVEF, LVFS, and CO, along with elevated LDH and CK-MB levels in cardiac enzyme profiles, confirming the successful establishment of the DIC model. Notably, simultaneous SII and Dox treatment significantly counteracted these changes. HE and Masson staining results further revealed that SII mitigated Dox-induced cardiomyocyte injury, myofiber breaks, and myocardial fibrosis, indicating its therapeutic potential against DIC. These findings suggest that SII can attenuate DIC in mice.

An excessive inflammatory response is crucial in DIC pathophysiology [18]. Dox activates NF-κB, leading to a rapid increase in pro-inflammatory cytokines such as IL-6, IL-1β, and TNF-α in cardiomyocytes [19]. This surge in pro-inflammatory mediators activates cell death pathways [20], culminating in cardiomyocyte apoptosis, compromised cardiac function, and potential heart failure. To investigate the role of SII in ameliorating Dox-induced myocardial injury through the modulation of inflammation, the levels of inflammatory markers in cardiomyocytes were analyzed. Western blot analysis and ELISA were used to quantify the expression levels of P-p65, IL-6, IL-1β, and TNF-α. The findings revealed significant upregulation of these inflammatory markers in the Dox group. Conversely, SII pretreatment reduced the levels of these inflammatory factors, suggesting that SII attenuates Dox-induced inflammatory responses in cardiomyocytes both *in vivo* and *in vitro*.

Oxidative stress is crucial in the pathogenesis of DIC [21], necessitating thorough investigation. MDA, a lipid peroxidation byproduct, signifies oxidative damage to cell membranes [22], whereas SOD and GSH-Px are essential antioxidant enzymes that mitigate oxidative stress by neutralizing free radicals [23]. Biochemical analyses revealed that Dox exposure significantly increased MDA levels and markedly decreased SOD and GSH-Px activities, indicating enhanced oxidative stress injury and reduced antioxidative defense in cardiac tissues. However, SII treatment effectively decreased the MDA content and restored SOD and GSH-Px activities, highlighting its protective role against oxidative stress. DHE staining was used to quantify the ROS levels in cardiomyocytes, further assessing oxidative stress. The results revealed a notable reduction in ROS accumulation in the Dox+SII group compared with the Dox group, underscoring the efficacy of SII in mitigating ROS-induced damage. To elucidate the molecular mechanisms underlying these observations, the expression levels of oxidative stress-related proteins, including Nrf2, HO-1, and NQO1, were analyzed through western blot analysis. Nrf2, a regulatory transcription factor activated by oxidative stress, promotes the expression levels of antioxidant proteins such as HO-1 and NQO1 [24]. HO-1 plays a protective role by catalyzing the breakdown of heme into antioxidant products [25], whereas NQO1 reduces free radical production through electron transfer reactions [26]. The results demonstrated that Dox exposure downregulated Nrf2 and its downstream antioxidant proteins, HO-1 and NQO1, in cardiomyocytes. Conversely, SII treatment significantly upregulated the expressions of these proteins, indicating an enhanced cellular antioxidant response and suggesting a mechanism by which SII attenuates Dox-induced oxidative stress.

Both inflammation and oxidative stress are critical pathways leading to apoptosis. Initially, inflammation triggers immune cells to secrete substantial quantities of inflammatory cytokines, such as TNF-α, IL-1β, and IL-6, which activate extrinsic and intrinsic apoptotic pathways [27]. Concurrently, ROS—including superoxide anions, hydrogen peroxide, and various free radicals—initiate lipid peroxidation on cell membranes [28], damaging unsaturated fatty acids, increasing membrane permeability, and leading to the leakage of cellular contents, culminating in apoptosis. Additionally, ROS damage DNA, proteins, carbohydrates, and other crucial intracellular molecules, prompting cells to commence apoptotic programs [29]. ROS can also disrupt normal cellular signaling by oxidatively modifying intracellular signaling molecules, thereby activating or inhibiting apoptosis-related pathways, including the P53, mitochondrial apoptosis, and MAPK pathways. The resulting damage from excessive inflammation and oxidative stress ultimately leads to apoptosis. In cardiomyocytes, excessive apoptosis can lead to cardiac dysfunction and potentially heart failure [30]. To investigate this, cardiomyocyte apoptosis in mice was assessed through TUNEL staining, flow cytometry, and western blot analysis. TUNEL staining revealed significant apoptosis in the cardiomyocytes of the Dox group, which was markedly reduced following SII treatment. Flow cytometry results demonstrated that SII could mitigate Dox-induced alterations in the MMP, thereby lowering the rate of apoptosis. Additionally, western blot analysis indicated that SII prevented the Dox-induced upregulation of pro-apoptotic proteins (BAX and cleaved caspase-3) and the downregulation of the antiapoptotic protein BCL-2. Collectively, these findings suggest that SII mitigates Dox-induced apoptosis and effectively ameliorates DIC.

Network pharmacology, an interdisciplinary field that combines bioinformatics with network biology, deciphers the molecular mechanisms of disease treatment by predicting interactions between components of traditional Chinese medicine and disease targets [ 31‒ 33]. In examining the effect of SII on DIC, network pharmacology approaches identified 43 common targets for the therapeutic action of SII. Analysis revealed that P53 is closely associated with cardiotoxicity, highlighting its critical role. GO enrichment and KEGG pathway analyses indicated that these targets primarily regulate cellular processes and respond to biological stimuli and are significantly associated with the P53 signaling pathway. Pathway enrichment analysis via the DAVID database suggested that the protective effect of SII against DIC involves modulating the AKT/MDM2/P53 signaling pathway. Notably, *P53* and *MDM2* emerged as the most crucial hub genes, ranking first and seventh, respectively.

The P53 protein regulates key cellular processes, including cell cycle arrest, DNA repair, apoptosis [34], and senescence. In cardiotoxicity, particularly in the context of Dox, the P53 pathway is vital. Dox induces complex damage to cardiomyocytes, leading to P53 activation as a damage response [35]. P53 activation halts cell proliferation and facilitates DNA repair and recovery [36]. When damage is extensive or metabolism is disrupted, P53 activation triggers apoptosis [37]. Although apoptosis removes damaged cells, excessive apoptosis in the heart leads to tissue degradation and loss of function, which are central to DIC [38]. Network pharmacological analysis suggested that SII mitigates DIC by modulating the AKT/MDM2/P53 signaling pathway. MDM2, an E3 ubiquitin ligase, usually targets P53 for degradation [39]. Dox disrupts this balance by inhibiting AKT signaling and reducing MDM2 phosphorylation, which decreases MDM2 activity toward P53 and increases P53 stability and activity, potentially leading to excessive apoptosis [40]. Modulating the AKT/MDM2/P53 pathway with SII could restore the balance between necessary P53-mediated DNA repair and prevent excessive apoptosis, preserving cardiac tissue integrity and function.

Western blot analysis was employed to validate the network pharmacology predictions by quantifying P-AKT, MDM2, and P53 protein expression levels in mouse heart tissues and H9c2 cell lines. The results indicated a notable decrease in P-AKT and MDM2 protein levels in the Dox group, alongside a significant increase in P53 protein expression. Conversely, SII treatment increased P-AKT and MDM2 protein levels while reducing P53 protein expression in cardiomyocytes. Nutlin-3a, an inhibitor of MDM2 and activator of P53, stabilizes P53 by inhibiting its interaction with MDM2, reducing P53 degradation. To further substantiate the underlying mechanism, additional experimental groups were treated with Nutlin-3a and a combination of Dox, SII, and Nutlin-3a (D+S+N). The groups included the Ctrl, SII, Nutlin-3a, Dox, Dox+SII, and D+S+N groups. Western blot analysis was used to quantify P-AKT, MDM2, P53, BAX, C-caspase3, and BCL-2 protein levels to elucidate the impact of Nutlin-3a pretreatment on the SII-mediated mitigation of DIC. Compared with the Dox + SII group, the D + S + N group presented significant increases in P53, BAX, and C-caspase3 protein levels and a notable decrease in BCL-2 protein level in H9c2 cells. Notably, P-AKT and MDM2 protein levels remained largely unchanged. These findings suggested that Nutlin-3a pretreatment inhibited MDM2-mediated P53 degradation, impairing the ability of SII to mitigate DIC *via* the AKT/MDM2/P53 pathway. These findings suggest that the protective effect of SII against DIC predominantly operates through the downregulation of the AKT/MDM2/P53 pathway.

This study demonstrated the capacity of SII to mitigate DIC. However, specific limitations were identified. First, the use of H9c2 cardiomyocytes derived from embryonic heart tissues contrasts with primary cardiomyocytes, which are typically isolated from neonatal mice, in terms of their proliferative capacity, mechanical attributes, and susceptibility to toxic damage [41]. Future *in vitro* investigations should prioritize primary cardiomyocytes to ensure greater relevance and applicability of the findings. Although primary cardiomyocytes were not used in our *in vitro* experiments, these observations were corroborated through phenotype and mechanism validation via animal experiments. Second, the *in vivo* experimental design employed a singular dosage regimen for SII (0.35 mg/kg/day) across both the SII and DOX+SII groups. To comprehensively evaluate the dose-dependent cardioprotective efficacy of SII against Dox-induced cardiac damage, future *in vivo* studies should incorporate an additional group in which Dox is administered in combination with a reduced concentration of SII. This adjustment will enable a more thorough exploration of the optimal dosage regimen for SII in mitigating DIC.

In conclusion, this study highlights the therapeutic potential of SII in mitigating cardiomyocyte injury induced by Dox-induced inflammation, oxidative stress, and apoptosis, as demonstrated through *in vivo* and *in vitro* experiments. Combined with network pharmacological analyses, the results suggested that SII has a significant protective effect against DIC in mice, which is likely related to the inhibition of the Dox-induced upregulation of the AKT/MDM2/P53 pathway. These insights not only bolster the groundwork for the clinical application of SII but also present a promising therapeutic strategy for alleviating DIC.
